# Spontaneous coronary artery dissection in a young man - Case report

**DOI:** 10.1186/1749-8090-6-22

**Published:** 2011-03-03

**Authors:** Julia Schmid, Johann Auer

**Affiliations:** 1 Department of Cardiology and Intensive Care, General Hospital Braunau, Austria, Ringstrasse 60, A - 5280 Braunau am Inn, Austria

## Abstract

A 31 year old man with a 17-year-history of drug abuse (heroine and cannabis) was admitted with recurrent chest pain over a period of about three weeks. Chest discomfort severely worsened during the 5 hours before hospital admission. Electrocardiography revealed poor R-wave progression and non specific repolarization abnormalities. Echocardiography showed extensive left ventricular anterior and apical wall motion abnormalities and a ventricular thrombus located at the apex of the left ventricle was present. Subsequently, a diagnosis of acute coronary syndrome was made. Coronary angiography revealed spontaneous coronary artery dissection of the left anterior descending (LAD) artery with Thrombolysis In Myocardial Infarction (TIMI) flow 2 to 3. We managed the patient conservatively. The clinical course was uneventful and repeated angiography on day 4 demonstrated spontaneous healing of large parts of the dissection with TIMI 3 flow in the LAD.

## Background

Spontaneous coronary artery dissection (SCAD) is a rare and uncommon case of sudden cardiac death and acute coronary syndrome [1]. As several diseases and conditions have been associated with SCAD it therefore probably constitutes a heterogeneous entity. Risk factors for SCAD comprise pregnancy, Ehlers-Danlos disease, Marfan's Syndrome, intensive exercise, or cocaine abuse [1-4]. The clinical presentation of SCAD depends on the extent and the flow limiting severity of the coronary artery dissection, and ranges from asymptomatic to unstable angina, acute myocardial infarction, ventricular arrhythmias to sudden cardiac death. Coronary angiography is frequently used in the evaluation of patients with acute coronary syndromes. Thus, most cases with SCAD are detected by angiography. Moreover, intracoronary imaging techniques such as intravascular ultrasound (IVUS) and optical coherence tomography (OCT), which provide detailed morphological information on coronary lesions and on the location of dissection planes between the different layers of the arterial wall, have enabled a more detailed clinical assessment of SCAD. Furthermore, non-invasive coronary angiography by multidetector computed tomography (MDCT) has been used for longitudinal follow-up evaluation of patients with SCAD. There is no consensus about the way of treatment including medical therapy, interventional treatment with PCI or surgery. We present a case of SCAD complicated by the occurrence of a left ventricular thrombus in a 31 years old man admitted with an acute coronary syndrome.

## Case report

**Figure 1:**
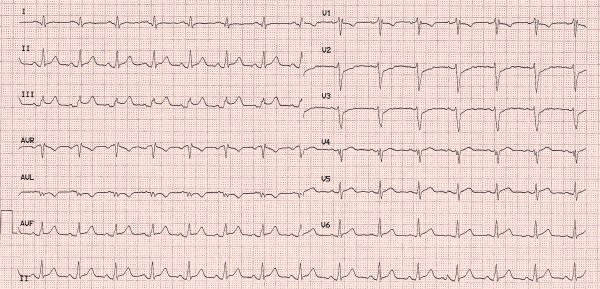
**Electrocardiogram at admission with poor R-wave progression and non specific repolarization abnormalities**.

**Figure 2:**
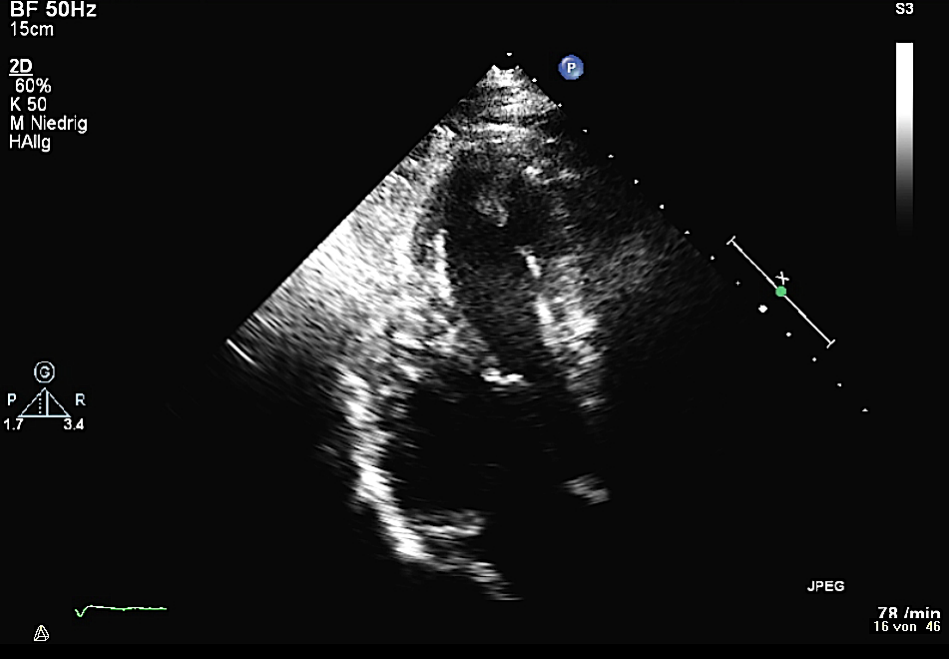
**Transthoracic echocardiography; 4 chamber view reveals left ventricular thrombus**.

**Figure 3:**
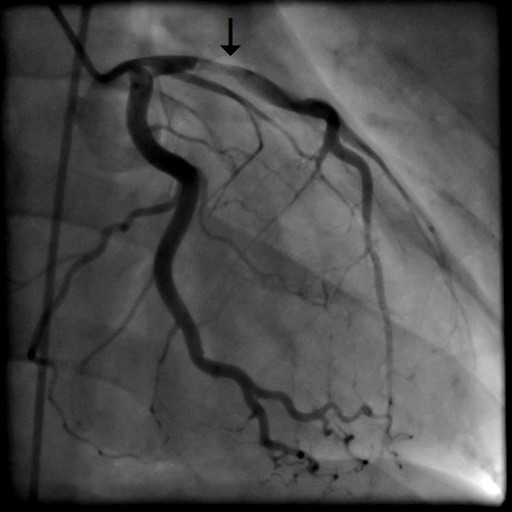
**Coronary angiography in RAO view with dissection of the left anterior descending artery**.

**Figure 4:**
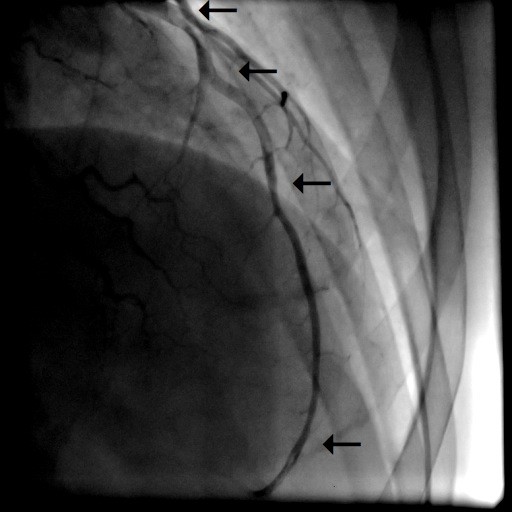
**Coronary angiography in posterior-anterior view with caudal angulation with dissection of the LAD**.

**Figure 5:**
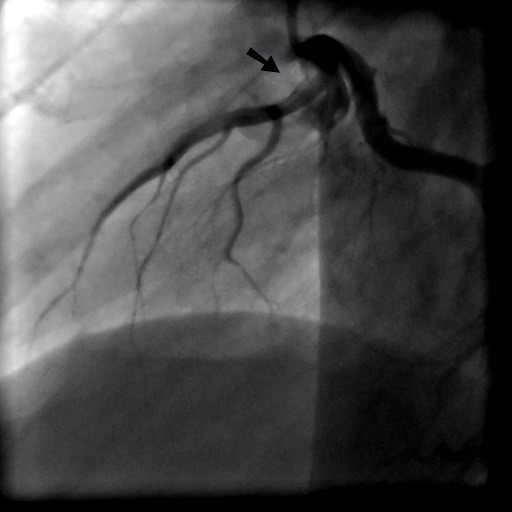
**Coronary angiography in LAO view with dissection of the left anterior descending artery**.

**Figure 6:**
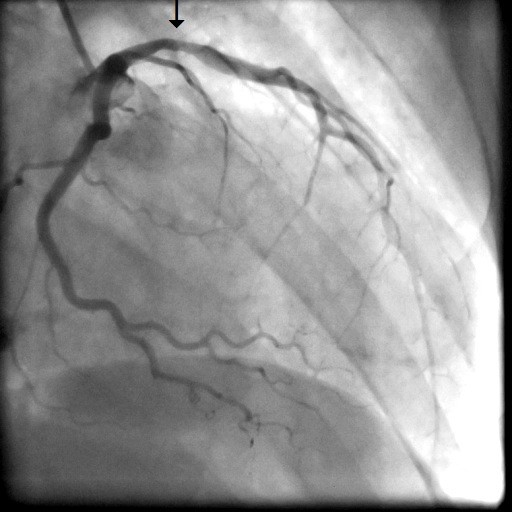
**Coronary angiography in RAO view 5 days after admission with dissection of the LAD**.

A 31-year old man was admitted to our intensive care unit with recurrent chest pain over a period of about three weeks. Chest discomfort severely worsened during the 5 hours before hospital admission. At admission the patient had severe chest pain. Physical examination of the chest did not reveal any abnormalities. Blood pressure at admission was 150/85 mmHg and pulse rate was 86 beats per minute. The medical history was remarkable for paranoid schizophrenia and mild anaemia resulting from iron deficiency. In addition, the patient had a history of drug (heroine, cannabis) and nicotine abuse for about 17 years. Three months ago, the patient suffered a stroke with vision disorders and a corresponding lesion at MR imaging. There sequelae persisted from this cerebrovascular accident. The family history revealed myocardial infarction of the father at the age of 65 years. Previous medication included clozapine 100 mg and benperidol 10 mg daily because of the history of paranoid schizophrenia and ferric sulphate because of anaemia. Electrocardiography (ECG) revealed sinus rhythm and poor R-wave progression and non specific repolarization abnormalities (Figure 1). Echocardiography showed extensive left ventricular anterior and apical wall motion abnormalities and a ventricular thrombus located at the apex of the left ventricle (Figure 2). Cardiac troponine I was 0,681 ng/ml (Abbott Laboratories, Illinois, U.S.A.; normal value < 0,032 ng/ml). The patient was treated with morphine hydrochloride, aspirin, clopidogrel, nitrates, bisoprolol, and unfractionated heparin for acute coronary syndrome. Based on the symptoms, ECG and echocardiographic findings and a positive cardiac biomarker, early coronary angiography was performed. The left anterior descending (LAD) artery showed extensive dissection with visible tear from the proximal part of the vessel to the apical LAD segment. The TIMI (thrombolysis in myocardial infarction) flow grade was 2+ (Figure 3, 4, 5). The right coronary artery (RCA) and the circumflex artery were normal. At the time of coronary angiography, chest pain had resolved completely. Based on the morphology of the vessel with an extensive dissection and TIMI II+ flow, we decided to manage this patient conservatively with close follow up. We continued unfractionated heparin to establish an activated partial thromboplastin time between 60 and 80 seconds (normal range 25 to 40 seconds), nitrates, dual antiplatelet therapy bisoprolol, and ramipril. On day 3 repeated coronary angiography showed a TIMI flow grade 3 in the LAD. The intimal tear was again visible with limited extent compared to the initial study. On day 5 we found no angiographically visible intimal tear any more. A diameter reduction of the proximal part of the LAD of about 40 to 50% persisted (Figure 6). The clinical course during hospital stay was uneventful. The patient could be discharged for cardiac rehabilitation 9 days after admission. Post-discharge treatment included dual antiplatelet therapy (aspirin 100 mg daily temporally unlimited, clopidogrel 75 mg daily for 12 months) in combination with phenprocoumone (international normalized ratio 2 to 3) for 3 months due to the left ventricular thrombus.

## Discussion

Spontaneous coronary artery dissection (SCAD) is a rare cause of acute coronary syndrome first described in 1931 [5]. The ratio female to men is 2:1 and the dissection is more frequently diagnosed in the left coronary artery [6]. Coronary artery dissection is characterized by a separation of the layers of the artery wall. This results in a false lumen or an intramural haematoma in the area of the media [2]. Coronary angiography is the primary tool for diagnosis of SCAD. Intracoronary imaging techniques such as intravascular ultrasound (IVUS) and optical coherence tomography (OCT), which provide detailed morphological information on coronary lesions and on the location of dissection planes between the different layers of the arterial wall, have enabled a more detailed clinical assessment of SCAD [2]. Furthermore, non-invasive coronary angiography by multidetector computed tomography (MDCT) has been used for longitudinal follow-up evaluation of patients with SCAD. We did not utilize IVUS in the patient presented in this report because angiographic assessment revealed high diagnostic accuracy. We did not expect further information from additional imaging that might have changed clinical decision making. SCAD occurs during pregnancy in 26,1% of the cases. In this patient population, SCAD was diagnosed most frequently during the postpartum period [7,8]. SCAD may be associated with Marfan's Syndrome, Ehlers-Danlos Disease, intensive exercise and cocaine abuse, female hormonal treatments as oral contraceptives, although in some cases no predictor could be identified [1-4]. A hereditary factor has been discussed previously [9]. There are no randomized trials on treatment of coronary artery dissection. The literature consists of case reports and case series. Different strategies of treatment have been discussed in the last years. Conservative management of patients with SCAD is a possible treatment strategy in stabile patients [10]. Antiplatelet therapy can be used because of the flow limitations caused by platelet thrombi [1]. GP IIb/IIIa inhibitors have been successfully used in patients with SCAD [2,11]. We did not use a GP IIb/IIIa inhibitor in the present patient because of clinical success with dual antiplatelet therapy and heparin and risk-benefit calculation with respect to the recent stoke. However, utilization of a GP IIb/IIIa inhibitor would have been our bail-out-strategy. Koller et al. reported a spontaneous healing of the lesion of a postpartum SCAD with the treatment including prednisone and cyclophosphamide combined with the conventional therapy [12]. Stent implantation can be performed in limited disease after identification of the true and false lumen [13]. Fibrinolysis is not recommended due to the increase of the bleeding risk [1]. In the case of multivessel dissection, coronary artery bypass graft (CABG) may be a reasonable choice [14]. In conclusion spontaneous coronary artery dissection is an uncommon disease, more frequently seen in women without cardiac risk factors [1]. The postpartum period, cocaine, intensive exercise and diseases like Ehlers-Danlos are risk factors for SCAD [1-4]. The management strategy has to be based on clinical presentation, additional findings and morphological details during invasive assessment in a case by case fashion.

## Consent

Written informed consent was obtained from the patient for publication of this case report and accompanying images. A copy of the written consent is available for review by the Editor-in-Chief of this journal.

## Competing interests

The authors declare that they have no competing interests.

## Authors' contributions

JS was the main author and wrote the article. JA was the cardiology consultant and gave final approval of the manuscript. All authors have read and approved the final manuscript.
